# 5-Fluorouracil response in a large panel of colorectal cancer cell lines is associated with mismatch repair deficiency

**DOI:** 10.1038/sj.bjc.6605780

**Published:** 2010-07-06

**Authors:** K Bracht, A M Nicholls, Y Liu, W F Bodmer

**Affiliations:** 1 Cancer Immunogenetics Laboratory, Weatherall Institute of Molecular Medicine, John Radcliffe Hospital, Oxford OX3 9DS, UK

**Keywords:** 5-fluorouracil, mismatch repair deficiency, replication error, colorectal cancer, cell lines

## Abstract

**Background:**

Colorectal cancer is (CRC) one of the commonest cancers and its therapy is still based on few drugs. Currently, no biological criteria are used to choose the most effective of the established drugs for treatment.

**Methods:**

A panel of 77 CRC cell lines was tested for sensitivity to 5-fluorouracil (5FU) using the SRB assay. The responses were grouped into three categories and correlated with genetic changes in the cell lines.

**Results:**

The strongest and most clearcut correlation was between 5-fluorouracil response and replication error status (mismatch repair deficiency). All the other significant correlations (loss of heterozygosity for *DCC* and mutations in *TGFbIIR*) are secondary to the association with replication error status.

**Interpretation and conclusion:**

Our findings validate previous analyses based mainly on clinical data, and indicate that replication error status could be a useful guide to 5-fluorouracil-based CRC therapy. Essentially, all previously described correlations with 5FU response are secondary to the association with replication error status.

Colorectal cancer (CRC) is one of the most commonly diagnosed cancers in the western world (Rim *et al*, 2009), and the 5-year relative survival rate is still only between 44 and 60% in the United Kingdom and North America (Coleman *et al*, 2008). A wide range of genetic changes due to mutations and loss of heterozygosity (LOH), as well as epigenetic changes, are found in CRC, indicating the considerable heterogeneity of the disease. Involved genes include *APC*, *KRAS*, *PIK3CA*, *BRAF*, *PTEN*, *SMAD4*, *MLH1*, *MSH2*, *TP53*, and *CTNNB1* (Sanger Institute). *KRAS* activating mutations have recently emerged as predictive biomarkers for treatment of CRC with EGFR inhibiting agents in clinical trials (Linardou *et al*, 2008; Riely and Ladanyi, 2008). Clinical data also suggest an association between 5-fluorouracil (5FU) response and RER status (Ribic *et al*, 2003; Warusavitarne and Schnitzler, 2007; Muller *et al*, 2008). These studies extended findings by previous work on cell-based studies in isogenic pairs of HCT116 cells (Meyers *et al*, 2001; Arnold *et al*, 2003). So far, however, the *KRAS* association is the only predictive marker that is being used to guide the treatment of CRC.

Advances in the chemotherapy treatment of CRC are limited by the currently available selection of licensed drugs, most of which (oxaliplatin, 5FU, irinotecan, and capecitabine) have been in use for many years. The most recently introduced agents are antibodies targeting EGFR (cetuximab and panitumumab) or VEGF (bevacizumab). 5-Fluorouracil, which is also used in the treatment of breast, stomach, and pancreatic cancer, remains the cornerstone of CRC treatment, although widely used in combination with several other drugs (Kopetz, 2008). It is an antimetabolite that, after conversion into its main active metabolites (FdÌMP, FdUTP, and FUTP), induces, among other effects, RNA and DNA damage through incorporation of its metabolites into nucleic acids, and inhibition of thymidylate synthase (TS) and therefore DNA synthesis (Longley *et al*, 2003). However, only 10–15% of advanced CRC tumours treated with 5FU/leucovorin first-line therapy respond (Longley *et al*, 2003; Chen *et al*, 2010), which highlights the need to establish predictive biomarkers for 5FU drug response.

We have accumulated in our laboratory a large collection of CRC-derived cell lines on which we have information regarding their genetic changes and variation in gene expression (see, e.g., Liu and Bodmer, 2006). This panel of cell lines provides a powerful tool for detecting associations between drug responses and the biological characteristics of the lines, which reflect the properties of the tumours from which they were derived. The use of the cell lines should, therefore, enable the discovery of new predictive markers for better patient selection and more targeted therapy.

In this study we present a robust method for large-scale screening of CRC cell lines directly from frozen stock and a simple objective approach to the classification of the cell lines with respect to their drug responses. Data from a screen of 5FU response in 77 cell lines were used for an analysis of correlations with genetic changes. The results show, in agreement with published clinical data, that the major genetic change affecting 5FU response is mismatch repair status, thus validating the appropriateness of our method of analysis.

## Materials and methods

### Cell lines

Details of the 77 cell lines used in this study are listed in Supplementary Table 1. Many of the lines have been described before (Liu and Bodmer, 2006). The cell lines were characterised with respect to mismatch repair deficiency (replication error status: RER+ for mismatch repair defective and RER− otherwise (four microsatellite loci, BAT25, BAT26, D17S250, and D18S69, were amplified and used to determine RER status; Efstathiou *et al*, 1999)), mutations in *APC* (mutation cluster region), *KRAS* (codons 12 and 13), *BRAF* (V600E), *TP53, CDH1* (E-cadherin), *CTNNB1* (*β*-catenin; exons 3, 4, 5, and 6), *MLH1*, *MSH2*, *PIK3CA* (exons 9 and 20), *B2M* (*β*-2-microglobulin, exons 1 and 2), *TGFBR2* (TGFbIIR), *CHK1,* and *SMAD4*, LOH around the genes *APC* and *CDX1* (chromosome 5), *SMAD4* and *DCC* (chromosome 18), and promoter methylation of *MLH1, MSH2, CDKN2A* (p16), *CDKN2B* (p15), and *CDX1*. Most cell lines have been HLA typed to identify duplicate cell lines, and all cell lines were routinely tested negative for mycoplasma.

### Toxicity testing

Increased throughput was predominately achieved by plating cells directly into 96-well plates from frozen stock, thus negating the need for continuous culture of the cell lines. Optimal freezing and thawing conditions were established and the time required, after plating, for lines to begin their growth phase was determined and defined as the lag time. Standard freezing conditions involved suspending cells in 5% DMSO in FCS at a density of 4 × 10^6^ cells per ml and placing them on dry ice for 90 min before transfer to liquid nitrogen. For use from frozen stocks, cells were warmed quickly to 37 °C and transferred immediately into prewarmed medium, centrifuged, and re-suspended in fresh, DMSO-free medium before use. Cell lines plated from thawed stocks were shown to have the same growth curves and drug dose-response curves as their cultured counterparts (data not shown). Lag times and doubling time (d.b.t.) for all cell lines after plating from frozen stock were established by plating 3000 cells per well and using the SRB assay as described below. The results are reported in Supplementary Table 1.

For toxicity testing, 3000 cells were plated in 100 *μ*l medium in 96-well plates and incubated for their lag time before drug treatment with a further 100 *μ*l of drug-containing medium in duplicate wells for each condition. Final concentrations of 5FU ranged from 0.13 to 100 *μ*M (1 : 3 titration) and the final concentration of DMSO in all wells was 0.067%. Cells were incubated with drug for 3 d.b.t. to accommodate for differences in their growth rates, before they were fixed and stained following a standard SRB protocol (Vichai and Kirtikara, 2006). In brief, after spinning plates for 10 min at 1500 r.p.m., 50 *μ*l of ice-cold trichloroacetic acid (2.6 M) was added and plates were incubated at 4 °C for 60 min. After washing the plates with 0.5 × PBSA, cells were stained with sulforhodamine B (0.04% in 1% acetic acid) for 30 min, washed with acetic acid (1%), and bound dye was dissolved in 200 *μ*l Tris (10 mM, pH 9.5) before measuring the OD at 540 nm in a plate reader. *T*/*C*_corr_ (%) (‘treated over control (corrected)’ with a correction for the OD of the number of cells at the time of treatment) is calculated as ((OD_drug, 3 d.b.t._−OD_*t*0_)/(OD_DMSO control, 3 d.b.t._−OD_*t*0_)) × 100, and GI_50_ values were calculated from the resulting dose-response curves using model 210 (dose-response one site, five-parameter logistic model, fit=(*A*+((*B*−*A*)/(1+*x*/*C*)^*D*))^*E*))), *A* and *B* unlocked) in XLfit (IDBS, Surrey, UK). The experiment was repeated two to four times for each cell line.

### Matrigel assay

A total of 700 cells were plated in 80 *μ*l Matrigel (basement membrane matrix, growth factor reduced, BD (Oxford, UK), no. 356231) in 96-well plates and treated with 0, 0.5, 5, and 50 *μ*M 5FU in duplicate wells each (150 *μ*l per well added on top of Matrigel/cells). The medium was exchanged for fresh drug-containing medium on days 3, 6, 9, and 12 before colonies were counted under a light microscope ( × 10 objective) on day 14. Three fields of vision (FOV) were counted per well, averaging counts per FOV over both wells, and the experiment was repeated three times. Four cell lines were chosen for their known different abilities to differentiate in this matrix: LS174T and HT29 (intermediate differentiation), and LS180 and SW1222 (well differentiated).

### Statistical analysis

The 77 cell lines were divided into terciles with respect to their sensitivity to 5FU: sensitive (*n*=26), intermediate response (*n*=26), and resistant (*n*=25). To correlate drug response with categorical data (e.g., mutation status), we used *χ*^2^ association and trend tests in 2 × 2 table and 3 × 2 tables for comparing categories of drug response with two genetic categories (e.g., wild type and mutant). For known duplicate cell lines, only one of each duplicate was counted for association studies. When drug response groupings differed between duplicate lines, both duplicates were omitted from the statistical analysis. Reported *P*-values were not corrected for multiple testing.

## Results

### 5FU drug response

**Figure 1:**
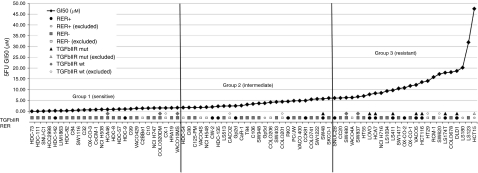
5-Fluorouracil GI_50_ values and correlation with RER status and TGFbIIR mutations. Sensitivity to 5FU is plotted by rank order. Cell lines are divided into three response groups (terciles). GI_50_ values=diamonds, RER status=circles (+) and squares (−), and mutations in *TGFbIIR*=triangles (mut) and diamonds (wt); cell lines from pairs of duplicates that are excluded from the analysis (see Materials and Methods) are represented by smaller and lighter symbols. Significance was tested as described in Table 1.

**Figure 2:**
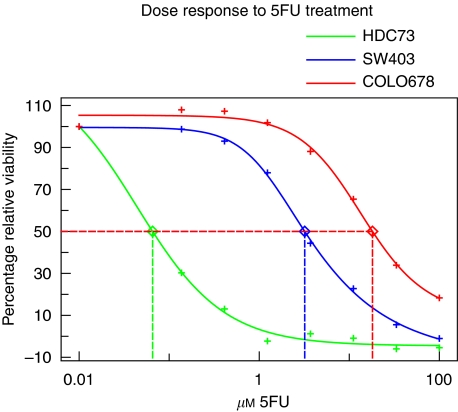
Representative dose-response curves. 5-Fluorouracil dose-response curves are shown using the SRB assay and incubation for three doubling times. Curves are examples from one experimental repeat and illustrate the response of the cell lines HDC73 (sensitive), SW403 (intermediate response), and COLO678 (resistant).

Overall, GI_50_ values ranged from 0.03 (HDC73) to 47.5 *μ*M (HCT15) in the 77 cell lines, giving an approximately 1600-fold maximum *μ*M difference. Supplementary Table 2 lists the GI_50_ values for all the cell lines, including duplicates, in alphabetical order and the same data are shown in rank order in Figure 1. Examples of dose-response curves for three cell lines with different levels of 5FU sensitivity are given in Figure 2. The cell lines were divided into three sensitivity groups, ranging from sensitive (HDC73 to VACO10MS, 0.03–1.70 *μ*M) to intermediate (HDC54 to SKCO1, 1.76–6.14 *μ*M) to resistant (SNUC2B to HCT15, 6.18–47.5 *μ*M), as shown in Figure 1. There is good agreement for the majority of pairs of duplicate cell lines. Thus, COLO201 and COLO206 (4.47 and 4.19 *μ*M), Gp2d and Gp5d (2.54 and 3.80 *μ*M), CACO2 and C2BBe1 (2.50 and 1.42 *μ*M), HDC54 and HDC57 (1.76 and 1.03 *μ*M), and LS174T and LS180 (17.87 and 20.20 *μ*M) agree well with each other. There is a greater difference between DLD1 and HCT15 (18.67 and 47.5 *μ*M), although these two cell lines have the fourth highest and highest GI_50_ values, respectively, and SW480 and SW620 (6.36 and 17.23 *μ*M), which were established, respectively, from the primary tumour and a metastasis. For all six pairs of these duplicate cell lines, both pairs fall into the same response category. The differences are larger for VACO4A and VACO4S (6.40 and 1.85 *μ*M), which were established from adherent and supernatant subclones of the same cell line. The generally good agreement between lines that have been separately maintained in culture for several years suggests a good measure of reproducibility in the drug response data that we have obtained, and gives confidence to the validity in the categorisation of the cell lines from sensitive to resistant.

### Drug responses in Matrigel

**Figure 3:**
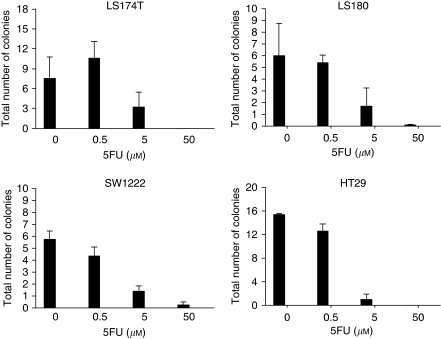
Drug responses in Matrigel. Total number of colonies per field of vision with a × 10 objective. s.d. are calculated from *n*=3 independent experiments.

Because this is not a higher throughput assay, only four cell lines (LS174T, LS180, SW1222, and HT29) were chosen for testing their sensitivity to 5FU in a 3D colony-forming assay using Matrigel. Two of these cell lines (SW1222 and LS180) readily form crypt-like structures, indicating their capacity to differentiate in Matrigel, whereas the other two (HT29 and LS174T) form crypt-like structures to a much lesser extent (Yeung *et al*, 2010). All four cell lines show a clear dose-response relationship (see Figure 3), with nearly complete inhibition of colony growth at the highest concentration of 5FU tested (50 *μ*M). There is no obvious relationship between the ability to form crypt-like structures and the 5FU response, suggesting that 5FU response is not related to the differentiation capacity of the lines. For all four cell lines, the concentration of 5FU that inhibits colony formation by 50% lies between 0.5 and 5 *μ*M 5FU. This suggests a somewhat higher sensitivity of the cell lines to 5FU in this clonogenicity assay than in our conventional SRB toxicity test, for which GI_50_ values ranged from 5.76 (SW1222) to 20.2 *μ*M (LS180).

### Correlation with genetic data

The classification of the lines into three sensitivity categories for 5FU enables a simple test for association between 5FU response and the genetic changes found in the cell lines. Visual inspection of Figure 1 immediately suggests a strong association between RER+ and 5FU resistance. This is confirmed by the statistical tests, excluding duplicate cell lines, shown in Table 1. The only significant associations among all the investigated parameters in 3 × 2 tables, testing for the association between the three categories of 5FU response and pairs of somatic genetic differences in the cell lines, are for RER+ *vs* RER− and for LOH around *DCC*, on chromosome 18. Mutations in *TGFbIIR,* which are nearly always found in microsatellite unstable tumours because of a coding mononucleotide repeat in the *TGFbIIR* gene (Markowitz *et al*, 1995; Samowitz *et al*, 2002), are on the margin of significance, with *P*=0.035. The LOH in SMAD4, also located on chromosome 18, misses significance with *P*=0.0759.

The LOH for chromosome 18 and mutations in *TGFbIIR* are well known to be associated with RER+ *vs* RER− status (Woodford-Richens *et al*, 2001), as also shown in our data in Table 2. This clearly implies that all these other associations are secondary to that with RER+ *vs* RER−.

We also found a significant correlation between the d.b.t. of cell lines and their sensitivity to 5FU in a *t*-test analysis, comparing the average 5FU GI_50_ differences between the slow and fast (d.b.t. >48 and ⩽48 h, respectively) growing cell lines (*P*=0.0005). Comparing cell lines’ growth rate by RER status also gives a significant result: the average d.b.t. for RER+ lines is 35.7 h, whereas that for RER− lines is longer (48.5 h, *P*=0.038, *t*-test) and this most probably accounts for the d.b.t. association with 5FU response.

## Discussion

Our key observation is that 5FU resistance in our panel of 77 CRC cell lines correlates strongly with mismatch repair deficiency, or RER+. These results are in agreement with the clinical data for the adjuvant setting (Ribic *et al*, 2003; Warusavitarne and Schnitzler, 2007; Muller *et al*, 2008), and hence support the use of a panel of cell lines for preclinical testing of associations of drug responses with genetic changes in cancers. As a recent meta-analysis by Des Guetz *et al* (2009) reported, the situation might be different in metastatic cancer, in which they found an overall hazard ratio (HR) of 0.83 for RER+ *vs* RER− patients treated with 5FU-based chemotherapy. It has to be noted, however, that HR ratios varied between 0.48 and 1.21 in the five studies selected by them.

When given no adjuvant chemotherapy, RER+ patients have an overall better prognosis(de la Chapelle, 2003; Ribic *et al*, 2003), as confirmed by a meta-analysis of 32 studies by Popat *et al* (2005). This may be explained by immune response to the mutant proteins that arise frequently in RER+ tumours because of frameshift mutations (Bodmer *et al*, 1994; Saeterdal *et al*, 2001). This suggestion is supported by the finding that RER+ tumours are characterised by a higher rate of lymphocyte infiltration (Buckowitz *et al*, 2005) that correlates with the total number of frameshift mutations (Tougeron *et al*, 2009). In addition, T cell-specific immune responses have been shown against MSI-induced frameshift neopeptides (Schwitalle *et al*, 2008). However, under 5FU-based chemotherapy, patients with microsatellite stable disease were found to have a significant increase in the duration of overall survival and disease-free survival, which was not observed for RER+ patients (Ribic *et al*, 2003; Carethers *et al*, 2004; Popat *et al*, 2005; Jover *et al*, 2006; Sargent *et al*, 2008).

Watanabe *et al* (2001) reported an improvement in 5-year overall survival for *TGFbIIR-*mutant patients under 5FU-based chemotherapy, but the results are controversial. A possible explanation for this discrepancy with other data and with our results lies in survival advantages gained from drugs other than 5FU used in the treatment. Our results were, of course, obtained under 5FU monotherapy.

The proportion of cell lines in our panel with mutations in MSH6 can be expected to be too low to show any significant correlations. This is also the case for MSH2, in which only three cell lines (LOVO, HCT15, and DLD1) were found to carry MSH2 mutations.

The well-recognised alterations in SMAD4 and DCC in RER+ CRCs and cell lines, as also shown in Table 2, translate into significant differences with respect to 5FU treatment (see Figure 1). This shows further that RER status is the major underlying determinant of 5FU sensitivity.

Following standard doses of 5FU given by intravenous continuous infusion, patient plasma levels reported in the literature lie in the range of 0.06–11.3 *μ*M (188-fold) (Poorter *et al*, 1995; Adjei *et al*, 2002), which overlaps the range of GI_50_ values observed in our cell line panel. This suggests that, under standard therapy, most tumours should have responded to 5FU to some extent, but the patients with RER+ tumours are likely to have responded much less than those with RER− tumours. Such patients should be identified and either given higher doses of 5FU, or not given a drug that is very unlikely to be of benefit for them.

Our data extend significantly previously published data on 5FU responses in cell lines. Thus, studies on a mixed panel of 14 cell lines showed a 31-fold variation in 5FU response (Bracht *et al*, 2006), when compared with the 1600-fold variation found in our study. Data from the NCI60 drug-screening programme (NSC 19893, 1806 experiments (DTP)) showed variation in GI_50_ values for 5FU response between 1.6 and 107.6 *μ*M in the limited number of CRC cell lines studied. The most extensive previous study on 5FU responses in 30 CRC lines showed a range of GI_50_ values between 0.7 and 23.1 *μ*M (Mariadason *et al*, 2003). An analysis of the ranked responses to 5FU in the 21 cell lines included both in Mariadason's *et al* (2003) and our study shows a significant correlation, giving *P*=0.0307 using the Wilcoxon signed-rank test. These results suggest reasonable reproducibility in the measures of 5FU response even when different techniques are used to assess the response, and lend weight to the use of cell lines for preclinical testing.

Our data agree with a much smaller study on three CRC cell lines undertaken by Carethers *et al* (1999), who excluded cell cycle alterations and differences in nucleotide uptake as a cause for their findings. The disagreement between our results and those of Mariadason *et al* (2003), who did not find RER status correlated with 5FU response, could be explained by differences in the size of the two cell line collections. Although the two panels contain similar proportions of RER+ cell lines (30 and 23.5%) our panel is more than twice the size (30 *vs* 77 lines with known RER status). We did not detect a correlation between p53 mutation status and 5FU response, which agrees with Mariadason *et al* (2003), who compared p53 with 5FU apoptotic response.

Although the link between mutations in *KRAS* and a response to anti-EGFR-based chemotherapy in CRC is well established, the clinical data regarding response to 5FU are more conflicting. Rosty *et al* (2001) found no significant difference between *KRAS* mutant and wild-type groups in the response of previously untreated CRC patients with liver metastasis to 5FU. Other researchers report reverse findings, with the 5FU single-agent response rate of 44% in *KRAS-*mutant patients *vs* only 32% in wild-type patients (Etienne-Grimaldi *et al*, 2008). However, the RASCAL study suggested that mutations in *KRAS* have a negative effect on survival rates and disease relapse (Russo *et al*, 2005). Our data do not show a significant correlation between *KRAS* mutation status and 5FU sensitivity (*P*=0.2713, data not shown). Analysis of preliminary data on TS mRNA expression and 5FU response suggests that high TS expression may be associated with resistance to 5FU. This has been suggested previously by others (Banerjee *et al*, 2002).

LS174T cells originate from trypsin treatment and LS180 cells from scraping of the primary culture from a tumour in the same patient (Rutzky *et al*, 1980). LS180 has been shown to express E-cadherin, whereas Kinsella *et al* (1994) found LS174T to be nonexpressing. This is explained by LS180 having just one mutation in the E-cadherin gene, whereas LS174T has a second mutation, leading to complete loss of E-cadherin expression in that cell line (Efstathiou *et al*, 1999 and David Bicknell, unpublished observations). Despite these differences, the cell lines show very similar responses to 5FU treatment, both in the Matrigel colony-forming assay and in SRB-measured response. This suggests that E-cadherin expression, and hence probably epithelial mesenchymal transition (EMT), does not correlate with 5FU response. The overall higher sensitivity of the four cell lines to 5FU in Matrigel compared with our conventional assay is most likely to be because of the difference between a colony-forming assay and a measure of cell number in bulk culture, such as the SRB assay. However, the data do suggest that there is no correlation between 5FU response and the capacity of the cell lines to differentiate. This, together with the lack of difference between LS180 and LS174T, suggests that the cancer stem cells and differentiated cells within the cell lines do not differ substantially in their response to 5FU.

There is now substantial evidence to support the view that cell lines are true representatives of the tumours from which they were derived. Thus, for example, both the frequency of specific mutations as well as their spectrum is similar between cell lines and tumour samples (see, e.g., Douglas *et al*, 2004; Sanger Institute). In addition, the availability of duplicate cell lines that have been separately maintained in culture for many years, and that in nearly all cases have the same genetic changes, shows that the genetic changes that we studied have not occurred in culture, and hence allows for internal quality control. The use of a large, disease-specific cell panel for drug response and related studies has several advantages: unlimited amounts of material are available, functional studies can be carried out, the size of the panel represents a wide variety of types and stages of CRC, and can help to uncover correlations that would not be observed with a smaller number of cell lines.

We suggest that our approach of studying the response to 5FU in a large panel of well-characterised cell lines, using a relatively high throughput test procedure and an objective categorisation of response, could be a model for preclinical testing of other drugs. Our results show that our approach can be a powerful tool for the detection of predictive markers for drug treatment responses, and that the cell line studies are good predictors of the clinical situation.

## Figures and Tables

**Table 1:** Correlation of 5FU response with genetic data

	**5FU**		
***P*=0.0022**	**1 (Sensitive)**	**2 (Intermediate)**	**3 (Resistant)**
*RER*			
+	1	6	9
−	22	13	11
			
	**5FU**		
***P*=0.035**	**1 (Sensitive)**	**2 (Intermediate) and 3 (resistant)**	
*TGFbIIR*			
mut	0	8	
wt	3	2	
			
	**5FU**		
***P*=0.0016**	**1 (Sensitive)**	**2 (Intermediate)**	**3 (Resistant)**
*DCC*			
LOH	10	8	5
No LOH	0	3	8

Abbreviations: 5FU=5-fluorouracil; LOH=loss of heterozygosity; RER=replication error. *P*-values for the *χ*^2^ trend test are given for the 3 × 2 tables, and a *P*-value for Fisher's exact test for the 2 × 2 table. Although the given *P*-values are not corrected for multiple testing, they remain significant for the RER+ *vs* RER− and LOH associations even when multiplied by 23 (Bonferroni correction), which is the number of such comparisons made. The only significant correlations are those shown in this table. Drug response was tested for correlations with RER status, mutations in *APC, kras, braf, TP53, E-cahderin (CDH1), β-catenin (CTNNB1), MLH1, MSH2, PIK3CA, β-2-microglobulin (B2M), TGFbIIR (TGFBR2), CHK1, SMAD4,* LOH of *APC, SMAD4, CDX1, DCC,* and *methylation of MLH1, MSH2, p16 (CDKN2A), p15 (CDKN2B),* and *CDX1*. Duplicate cell lines were excluded from the analysis when they fell into different 5FU response groups and one of the duplicate lines was included per pair when they fell into the same response category.

**Table 2:** Correlation between genetic variables and RER status

	***TGFbIIR***	
***P*=0.003**	**wt**	**mut**
*RER*		
+	1	8
−	5	0
		
	***DCC***	
***P*<0.0001**	**No LOH**	**LOH**
*RER*		
+	9	0
−	2	23

Abbreviations: LOH=loss of heterozygosity; RER=replication error. The associations between RER status and mutations in *TGFbIIR* (*n*=14) and LOH in *DCC* (*n*=33) when only one of the duplicate cell lines is included for each pair of duplicates are highly significant. The *P*-values are for Fisher's exact test in 2 × 2 contingency tables.

## References

[bib1] Adjei AA, Reid JM, Diasio RB, Sloan JA, Smith DA, Rubin J, Pitot HC, Alberts SR, Goldberg RM, Hanson LJ, Atherton P, Ames MM, Erlichman C (2002) Comparative pharmacokinetic study of continuous venous infusion fluorouracil and oral fluorouracil with eniluracil in patients with advanced solid tumors. J Clin Oncol 20: 1683–1691 11896120 10.1200/JCO.2002.20.6.1683

[bib2] Arnold CN, Goel A, Boland CR (2003) Role of hMLH1 promoter hypermethylation in drug resistance to 5-fluorouracil in colorectal cancer cell lines. Int J Cancer 106: 66–73 12794758 10.1002/ijc.11176

[bib3] Banerjee D, Mayer-Kuckuk P, Capiaux G, Budak-Alpdogan T, Gorlick R, Bertino JR (2002) Novel aspects of resistance to drugs targeted to dihydrofolate reductase and thymidylate synthase. Biochim Biophys Acta 1587: 164–173 12084458 10.1016/s0925-4439(02)00079-0

[bib4] Bodmer W, Bishop T, Karran P (1994) Genetic steps in colorectal cancer. Nat Genet 6: 217–219 8012377 10.1038/ng0394-217

[bib5] Bracht K, Boubakari, Grunert R, Bednarski PJ (2006) Correlations between the activities of 19 anti-tumor agents and the intracellular glutathione concentrations in a panel of 14 human cancer cell lines: comparisons with the National Cancer Institute data. Anticancer Drugs 17: 41–51 16317289 10.1097/01.cad.0000190280.60005.05

[bib6] Buckowitz A, Knaebel HP, Benner A, Blaker H, Gebert J, Kienle P, von Knebel Doeberitz M, Kloor M (2005) Microsatellite instability in colorectal cancer is associated with local lymphocyte infiltration and low frequency of distant metastases. Br J Cancer 92: 1746–1753 15856045 10.1038/sj.bjc.6602534 PMC2362037

[bib7] Carethers JM, Chauhan DP, Fink D, Nebel S, Bresalier RS, Howell SB, Boland CR (1999) Mismatch repair proficiency and *in vitro* response to 5-fluorouracil. Gastroenterology 117: 123–131 10381918 10.1016/s0016-5085(99)70558-5 PMC4343206

[bib8] Carethers JM, Smith EJ, Behling CA, Nguyen L, Tajima A, Doctolero RT, Cabrera BL, Goel A, Arnold CA, Miyai K, Boland CR (2004) Use of 5-fluorouracil and survival in patients with microsatellite-unstable colorectal cancer. Gastroenterology 126: 394–401 14762775 10.1053/j.gastro.2003.12.023

[bib9] Chen ML, Fang CH, Liang LS, Dai LH, Wang XK (2010) A meta-analysis of chemotherapy regimen fluorouracil/leucovorin/oxaliplatin compared with fluorouracil/leucovorin in treating advanced colorectal cancer. Surg Oncol 19: 38–45 19345093 10.1016/j.suronc.2009.02.015

[bib10] Coleman MP, Quaresma M, Berrino F, Lutz JM, De Angelis R, Capocaccia R, Baili P, Rachet B, Gatta G, Hakulinen T, Micheli A, Sant M, Weir HK, Elwood JM, Tsukuma H, Koifman S, GA ES, Francisci S, Santaquilani M, Verdecchia A, Storm HH, Young JL (2008) Cancer survival in five continents: a worldwide population-based study (CONCORD). Lancet Oncol 9: 730–756 18639491 10.1016/S1470-2045(08)70179-7

[bib11] de la Chapelle A (2003) Microsatellite instability. N Engl J Med 349: 209–210 12867603 10.1056/NEJMp038099

[bib12] Des Guetz G, Uzzan B, Nicolas P, Schischmanoff O, Morere JF (2009) Microsatellite instability: a predictive marker in metastatic colorectal cancer? Target Oncol 4: 57–62 19343302 10.1007/s11523-008-0103-8

[bib13] Douglas EJ, Fiegler H, Rowan A, Halford S, Bicknell DC, Bodmer W, Tomlinson IP, Carter NP (2004) Array comparative genomic hybridization analysis of colorectal cancer cell lines and primary carcinomas. Cancer Res 64: 4817–4825 15256451 10.1158/0008-5472.CAN-04-0328

[bib14] DTP NSC 19893. http://dtp.nci.nih.gov/dtpstandard/servlet/MeanGraphSummary?testshortname=NCI+Cancer+Screen+Current+Data&searchtype=NSC&searchlist=19893 (accessed 7 May 2010)

[bib15] Efstathiou JA, Liu D, Wheeler JM, Kim HC, Beck NE, Ilyas M, Karayiannakis AJ, Mortensen NJ, Kmiot W, Playford RJ, Pignatelli M, Bodmer WF (1999) Mutated epithelial cadherin is associated with increased tumorigenicity and loss of adhesion and of responsiveness to the motogenic trefoil factor 2 in colon carcinoma cells. Proc Natl Acad Sci USA 96: 2316–2321 10051639 10.1073/pnas.96.5.2316 PMC26781

[bib16] Etienne-Grimaldi MC, Formento JL, Francoual M, Francois E, Formento P, Renee N, Laurent-Puig P, Chazal M, Benchimol D, Delpero JR, Letoublon C, Pezet D, Seitz JF, Milano G (2008) K-Ras mutations and treatment outcome in colorectal cancer patients receiving exclusive fluoropyrimidine therapy. Clin Cancer Res 14: 4830–4835 18676755 10.1158/1078-0432.CCR-07-4906

[bib17] Jover R, Zapater P, Castells A, Llor X, Andreu M, Cubiella J, Pinol V, Xicola RM, Bujanda L, Rene JM, Clofent J, Bessa X, Morillas JD, Nicolas-Perez D, Paya A, Alenda C (2006) Mismatch repair status in the prediction of benefit from adjuvant fluorouracil chemotherapy in colorectal cancer. Gut 55: 848–855 16299036 10.1136/gut.2005.073015 PMC1856227

[bib18] Kinsella AR, Lepts GC, Hill CL, Jones M (1994) Reduced E-cadherin expression correlates with increased invasiveness in colorectal carcinoma cell lines. Clin Exp Metastasis 12: 335–342 8039307 10.1007/BF01753841

[bib19] Kopetz S (2008) Targeted therapy in colorectal cancer. In Targeted Cancer Therapy, Kurzrock R, Markman M (eds), 101. Humana Press: Totowa, NJ, USA

[bib20] Linardou H, Dahabreh IJ, Kanaloupiti D, Siannis F, Bafaloukos D, Kosmidis P, Papadimitriou CA, Murray S (2008) Assessment of somatic k-RAS mutations as a mechanism associated with resistance to EGFR-targeted agents: a systematic review and meta-analysis of studies in advanced non-small-cell lung cancer and metastatic colorectal cancer. Lancet Oncol 9: 962–972 18804418 10.1016/S1470-2045(08)70206-7

[bib21] Liu Y, Bodmer WF (2006) Analysis of P53 mutations and their expression in 56 colorectal cancer cell lines. Proc Natl Acad Sci USA 103: 976–981 16418264 10.1073/pnas.0510146103 PMC1327731

[bib22] Longley DB, Harkin DP, Johnston PG (2003) 5-Fluorouracil: mechanisms of action and clinical strategies. Nat Rev 3: 330–338 10.1038/nrc1074 12724731

[bib23] Mariadason JM, Arango D, Shi Q, Wilson AJ, Corner GA, Nicholas C, Aranes MJ, Lesser M, Schwartz EL, Augenlicht LH (2003) Gene expression profiling-based prediction of response of colon carcinoma cells to 5-fluorouracil and camptothecin. Cancer Res 63: 8791–8812 14695196

[bib24] Markowitz S, Wang J, Myeroff L, Parsons R, Sun L, Lutterbaugh J, Fan RS, Zborowska E, Kinzler KW, Vogelstein B, Brattain M, Willson JKV (1995) Inactivation of the type II TGF-beta receptor in colon cancer cells with microsatellite instability. Science (New York, NY) 268: 1336–1338 10.1126/science.7761852 7761852

[bib25] Meyers M, Wagner MW, Hwang HS, Kinsella TJ, Boothman DA (2001) Role of the hMLH1 DNA mismatch repair protein in fluoropyrimidine-mediated cell death and cell cycle responses. Cancer Res 61: 5139–5201 11431359

[bib26] Muller CI, Schulmann K, Reinacher-Schick A, Andre N, Arnold D, Tannapfel A, Arkenau H, Hahn SA, Schmoll SH, Porschen R, Schmiegel W, Graeven U (2008) Predictive and prognostic value of microsatellite instability in patients with advanced colorectal cancer treated with a fluoropyrimidine and oxaliplatin containing first-line chemotherapy. A report of the AIO Colorectal Study Group. Int J Colorectal Dis 23: 1033–1039 18594845 10.1007/s00384-008-0504-2

[bib27] Poorter RL, Peters GJ, Bakker PJ, Taat CW, Biermans-van Leeuwe DM, Codacci-Pisanelli G, Noordhuis P, Oosting J, Veenhof CH (1995) Intermittent continuous infusion of 5-fluorouracil and low dose oral leucovorin in patients with gastrointestinal cancer: relationship between plasma concentrations and clinical parameters. Eur J Cancer 31A: 1465–1470 7577073 10.1016/0959-8049(95)00217-7

[bib28] Popat S, Hubner R, Houlston RS (2005) Systematic review of microsatellite instability and colorectal cancer prognosis. J Clin Oncol 23: 609–618 15659508 10.1200/JCO.2005.01.086

[bib29] Ribic CM, Sargent DJ, Moore MJ, Thibodeau SN, French AJ, Goldberg RM, Hamilton SR, Laurent-Puig P, Gryfe R, Shepherd LE, Tu D, Redston M, Gallinger S (2003) Tumor microsatellite-instability status as a predictor of benefit from fluorouracil-based adjuvant chemotherapy for colon cancer. N Engl J Med 349: 247–257 12867608 10.1056/NEJMoa022289 PMC3584639

[bib30] Riely GJ, Ladanyi M (2008) KRAS mutations: an old oncogene becomes a new predictive biomarker. J Mol Diagn 10: 493–495 18832458 10.2353/jmoldx.2008.080105 PMC2570631

[bib31] Rim SH, Seeff L, Ahmed F, King JB, Coughlin SS (2009) Colorectal cancer incidence in the United States, 1999–2004: an updated analysis of data from the National Program of Cancer Registries and the Surveillance, Epidemiology, and End Results Program. Cancer 115: 1967–1976 19235249 10.1002/cncr.24216

[bib32] Rosty C, Chazal M, Etienne MC, Letoublon C, Bourgeon A, Delpero JR, Pezet D, Beaune P, Laurent-Puig P, Milano G (2001) Determination of microsatellite instability, p53 and K-RAS mutations in hepatic metastases from patients with colorectal cancer: relationship with response to 5-fluorouracil and survival. Int J Cancer 95: 162–167 11307149 10.1002/1097-0215(20010520)95:3<162::aid-ijc1028>3.0.co;2-j

[bib33] Russo A, Bazan V, Agnese V, Rodolico V, Gebbia N (2005) Prognostic and predictive factors in colorectal cancer: Kirsten Ras in CRC (RASCAL) and TP53CRC collaborative studies. Ann Oncol 16(Suppl 4): iv44–iv49 15923428 10.1093/annonc/mdi907

[bib34] Rutzky LP, Kaye CI, Siciliano MJ, Chao M, Kahan BD (1980) Longitudinal karyotype and genetic signature analysis of cultured human colon adenocarcinoma cell lines LS180 and LS174T. Cancer Res 40: 1443–1448 7370982

[bib35] Saeterdal I, Bjorheim J, Lislerud K, Gjertsen MK, Bukholm IK, Olsen OC, Nesland JM, Eriksen JA, Moller M, Lindblom A, Gaudernack G (2001) Frameshift-mutation-derived peptides as tumor-specific antigens in inherited and spontaneous colorectal cancer. Proc Natl Acad Sci USA 98: 13255–13260 11687624 10.1073/pnas.231326898 PMC60857

[bib36] Samowitz WS, Curtin K, Neuhausen S, Schaffer D, Slattery ML (2002) Prognostic implications of BAX and TGFBRII mutations in colon cancers with microsatellite instability. Genes, Chromosomes Cancer 35: 368–371 12378532 10.1002/gcc.10125

[bib37] Sanger Institute. Cosmic Database, www.sanger.ac.uk/genetics (accessed 7 May 2010)

[bib38] Sargent DJ, Marsoni S, Thibodeau SN, Labianca R, Hamilton SR, Torri V, Monges G, Ribic C, Grothey A, Gallinger S (2008) Confirmation of deficient mismatch repair (dMMR) as a predictive marker for lack of benefit from 5-FU based chemotherapy in stage II and III colon cancer (CC): a pooled molecular reanalysis of randomized chemotherapy trials. J Clin Oncol 26: 4008

[bib39] Schwitalle Y, Kloor M, Eiermann S, Linnebacher M, Kienle P, Knaebel HP, Tariverdian M, Benner A, von Knebel Doeberitz M (2008) Immune response against frameshift-induced neopeptides in HNPCC patients and healthy HNPCC mutation carriers. Gastroenterology 134: 988–997 18395080 10.1053/j.gastro.2008.01.015

[bib40] Tougeron D, Fauquembergue E, Rouquette A, Le Pessot F, Sesboue R, Laurent M, Berthet P, Mauillon J, Di Fiore F, Sabourin JC, Michel P, Tosi M, Frebourg T, Latouche JB (2009) Tumor-infiltrating lymphocytes in colorectal cancers with microsatellite instability are correlated with the number and spectrum of frameshift mutations. Mod Pathol 22: 1186–1195 19503063 10.1038/modpathol.2009.80

[bib41] Vichai V, Kirtikara K (2006) Sulforhodamine B colorimetric assay for cytotoxicity screening. Nat Protoc 1: 1112–1116 17406391 10.1038/nprot.2006.179

[bib42] Warusavitarne J, Schnitzler M (2007) The role of chemotherapy in microsatellite unstable (MSI-H) colorectal cancer. Int J Colorectal Dis 22: 739–748 17109103 10.1007/s00384-006-0228-0

[bib43] Watanabe T, Wu TT, Catalano PJ, Ueki T, Satriano R, Haller DG, Benson III AB, Hamilton SR (2001) Molecular predictors of survival after adjuvant chemotherapy for colon cancer. N Engl J Med 344: 1196–1206 11309634 10.1056/NEJM200104193441603 PMC3584633

[bib44] Woodford-Richens KL, Rowan AJ, Gorman P, Halford S, Bicknell DC, Wasan HS, Roylance RR, Bodmer WF, Tomlinson IP (2001) SMAD4 mutations in colorectal cancer probably occur before chromosomal instability, but after divergence of the microsatellite instability pathway. Proc Natl Acad Sci USA 98: 9719–9723 11481457 10.1073/pnas.171321498 PMC55519

[bib45] Yeung TM, Gandhi SC, Wilding JL, Muschel R, Bodmer WF (2010) Cancer stem cells from colorectal cancer-derived cell lines. Proc Natl Acad Sci USA 107: 3722–3727 20133591 10.1073/pnas.0915135107 PMC2840416

