# Supplementary material for: 5-Fluorouracil response in a large panel of colorectal cancer cell lines is associated with mismatch repair deficiency
Source: Br J Cancer. 2010 Jul 6;103(3):340–6. doi: 10.1038/sj.bjc.6605780 (PMC2920028; doi:10.1038/sj.bjc.6605780)
Supplement: Supplementary Table 2 [file 6605780x2.doc]

SI table 2

| **cell line** | **average GI_50_ (uM)** | **SD** |  | **cell line** | **average GI_50_ (uM)** | **SD** |  | **cell line** | **average GI_50_ (uM)** | **SD** |
| --- | --- | --- | --- | --- | --- | --- | --- | --- | --- | --- |
| C10 | 1.49 | 0.57 |  | HCT116 | 13.50 | 2.41 |  | OX-CO-1 | 11.75 | 1.55 |
| C106 | 3.45 | 1.19 |  | HCT15 | 47.50 | 12.40 |  | OX-CO-2 | 10.86 | 5.32 |
| C125-PM | 1.79 | 1.02 |  | HDC-111 | 0.05 | 0.00 |  | OX-CO-3 | 0.68 | 0.13 |
| C2BBe1 | 1.42 | 0.36 |  | HDC-135 | 2.36 | 0.09 |  | PC/JW | 4.90 | 0.42 |
| C32 | 0.65 | 0.07 |  | HDC-142 | 0.28 | 0.13 |  | RCM-1 | 15.83 | 3.10 |
| C80 | 1.78 | 0.03 |  | HDC-54 | 1.76 | 0.86 |  | RKO | 4.60 | 0.55 |
| C84 | 0.42 | 0.07 |  | HDC-57 | 1.03 | 0.09 |  | SKCO-1 | 6.14 | 3.46 |
| C99 | 1.13 | 0.47 |  | HDC-73 | 0.03 | 0.01 |  | SNU-C1 | 0.09 | 0.03 |
| CACO2 | 2.50 | 0.64 |  | HDC-8 | 0.93 | 0.39 |  | SNUC2B | 6.18 | 0.54 |
| CaR-1 | 2.70 | 1.29 |  | HDC-82 | 0.30 | 0.10 |  | SW1116 | 0.53 | 0.64 |
| CC20 | 6.20 | 1.49 |  | HDC-9 | 1.05 | 0.35 |  | SW1222 | 5.76 | 1.35 |
| CCK81 | 4.99 | 0.31 |  | HRA19 | 1.62 | 0.73 |  | SW1417 | 10.57 | 3.95 |
| CoCM-1 | 0.80 | 0.20 |  | HT29 | 14.05 | 5.02 |  | SW403 | 4.37 | 1.69 |
| COLO201 | 4.47 | 2.23 |  | HT55 | 7.10 | 1.44 |  | SW48 | 5.81 | 1.30 |
| COLO206 | 4.19 | 2.39 |  | LIM1863 | 0.29 | 0.12 |  | SW480 | 6.36 | 1.54 |
| COLO320DM | 1.57 | 0.43 |  | LOVO | 7.87 | 1.22 |  | SW620 | 17.23 | 2.06 |
| COLO678 | 18.10 | 0.01 |  | LS1034 | 9.41 | 0.14 |  | SW837 | 6.79 | 1.78 |
| COLO741 | 5.55 | 1.87 |  | LS123 | 31.94 | 33.35 |  | SW948 | 3.71 | 0.28 |
| CW-2 | 2.06 | 1.35 |  | LS174T | 17.87 | 3.57 |  | T84 | 3.18 | 3.45 |
| CX-1 | 1.57 | 0.35 |  | LS180 | 20.20 | 3.28 |  | VACO 400 | 4.93 | 1.98 |
| DLD1 | 18.67 | 6.93 |  | LS411 | 9.77 | 4.83 |  | VACO10MS | 1.70 | 0.86 |
| Gp2d | 2.54 | 1.27 |  | LS513 | 2.47 | 0.49 |  | VACO429 | 1.40 | 0.21 |
| Gp5d | 3.80 | 4.42 |  | NCI H508 | 0.89 | 1.34 |  | VACO4A | 6.40 | 2.48 |
| HCA46 | 0.89 | 0.44 |  | NCI H548 | 1.96 | 0.00 |  | VACO4S | 1.85 | 2.41 |
| HCA7 | 8.39 | 2.64 |  | NCI H716 | 8.52 | 1.64 |  | VACO5 | 12.21 | 1.27 |
| HCC2998 | 0.19 | 0.06 |  | NCI H747 | 1.55 | 1.04 |  |  |  |  |
