# Supplementary material for: 5-Fluorouracil response in a large panel of colorectal cancer cell lines is associated with mismatch repair deficiency
Source: Br J Cancer. 2010 Jul 6;103(3):340–6. doi: 10.1038/sj.bjc.6605780 (PMC2920028; doi:10.1038/sj.bjc.6605780)
Supplement: Supplementary Table Legends [file 6605780x3.doc]

***Supplementary Information Table 1: List of 85 cell lines***

List of all 77 cell lines included in the study. Duplicate cell lines are coded with the same colour. Detailed are the culture medium, method of detachment, lag time (days) after plating from frozen stock, doubling time (hours) in 96 well plates, RER status (with reference) and source of the cell line. Lag time and doubling time were established only once and in the same experiment. Medium was supplied from CRUK and Invitrogen, 10 % FCS (unless stated otherwise) and 1:100 penicillin/streptomycin (Invitrogen) and glutamine (if not contained in medium; CRUK) were added to the medium. Cells cultured in IMDM and EMDM were cultivated at 10 % CO_2_ while RPMI1640 cultures are incubated at 5 % CO_2_ (unless otherwise stated). 0.5 % Trypsin with EDTA diluted in PBSA were used for detachment. “Ca^2+^ depletion” describes cell dissociation via overnight incubation with SMEM medium (Invitrogen, low in calcium) while “EDTA only” uses EDTA alone to detach cells from the culture flasks. Cell lines growing in suspension are passaged by dilution in fresh culture medium.

***Supplementary Information Table 2: 5FU GI_50_ values***

Alphabetical list of GI_50_ values for all 77 cell lines. Standard deviation is calculated from n=2-4 independent experiments.

are calculated from n=3 independent experiments.
